# Cxcl14 depletion accelerates skeletal myogenesis by promoting cell cycle withdrawal

**DOI:** 10.1038/npjregenmed.2016.17

**Published:** 2017-01-05

**Authors:** Rachel J Waldemer-Streyer, Adriana Reyes-Ordoñez, Dongwook Kim, Rongping Zhang, Nilmani Singh, Jie Chen

**Affiliations:** 1 Department of Cell and Developmental Biology, University of Illinois at Urbana-Champaign, Urbana, IL, USA

## Abstract

Skeletal muscle in adults retains a robust ability to regenerate after injury, which progressively declines with age. Many of the regulators of skeletal myogenesis are unknown or incompletely understood. Intriguingly, muscle cells secrete a wide variety of factors, such as cytokines, which can influence muscle development and regeneration in an autocrine or paracrine manner. Here we describe chemokine (C-X-C motif) ligand 14 (Cxcl14) as a novel negative regulator of skeletal myogenesis. We found that Cxcl14 expression in myoblasts prevented cell cycle withdrawal, thereby inhibiting subsequent differentiation. Knockdown of Cxcl14 *in vitro* enhanced myogenic differentiation through promoting cell cycle withdrawal in an ERK1/2-dependent manner. Recapitulating these *in vitro* observations, the process of muscle regeneration following injury in young adult mice was accelerated by Cxcl14 depletion, accompanied by reduced cell proliferation. Furthermore, impaired capacity for muscle regeneration in aging mice was fully restored by Cxcl14 depletion. Our results indicate that Cxcl14 may be a promising target for development of therapeutics to treat muscle disease, especially aging-related muscle wasting.

## Introduction

Mature skeletal muscle tissue contains a resident population of stem cells that allows for a great capacity to regenerate. These satellite cells exist in a quiescent state under the basal lamina of myofibers until stimulated to divide by muscular injury.^1^ Effective myogenesis depends on the daughter myoblasts successfully completing a well-ordered series of processes, including withdrawal from the cell cycle, expression of many of the same myogenic genes seen in embryonic development, such as the MEF2 and MyoD families of transcription factors,^2^ and morphological changes and myoblast fusion that ultimately result in formation of multinucleated myofibers.^3^ The steps involved in skeletal myogenesis are well-conserved across species.^3^ However, the complex signalling mechanisms underlying those steps remain incompletely understood.

Recent studies have suggested that muscle cell-secreted proteins such as cytokines and growth factors may have an under-appreciated role in modulating muscle development and regeneration. One such study identified a large number of chemokine mRNAs that are differentially expressed by myoblasts at various time points during differentiation.^4^ Analyses of the muscle cell secretome corroborate this finding at the protein level.^5–7^ An unbiased functional screen of mouse cytokines revealed potential muscle-derived regulators of myogenesis belonging to distinct functional groups^8^ and some of them have since been demonstrated to have important roles in muscle development both *in vitro* and *in vivo*.^9,10^

Despite their necessary role in a variety of physiological processes, dysregulation of cytokines has long been associated with human diseases, especially those that involve acute or chronic inflammation. The physiological function of chemokine (C-X-C motif) ligand 14 (Cxcl14) is poorly understood. Also known as BRAK (breast and kidney-expressed chemokine), Cxcl14 expression is relatively high in many normal tissues but lost in various types of malignancies.^11,12^ Conversely, there are a number of reports demonstrating Cxcl14 overexpression in various tumour microenvironments, and implicating Cxcl14 in invasion and metastasis,^13–16^ indicating that its role in cancer may be cell-type specific.

Cxcl14 has also been linked to obesity and insulin resistance,^17,18^ and it may be involved in the immune response, as it has antimicrobial activity against respiratory tract bacteria and opportunistic skin pathogens.^19,20^ Cxcl14 has chemotactic activities *in vitro* for some immune cells.^21–24^ However, a Cxcl14^−/−^ mouse line displayed no deficiencies in activation, migration or peripheral tissue recruitment of monocytes, macrophages, dendritic cells, Langerhans cells or lymphocytes.^25^ Our current study reveals Cxcl14 as a negative regulator of skeletal muscle regeneration through its role in cell cycle progression. To the best of our knowledge, this is the first report of Cxcl14 function in muscle development or in regeneration of any tissue type.

## Results

### Cxcl14 expression is regulated during skeletal myogenesis *in vitro* and *in vivo*

**Figure 1:**
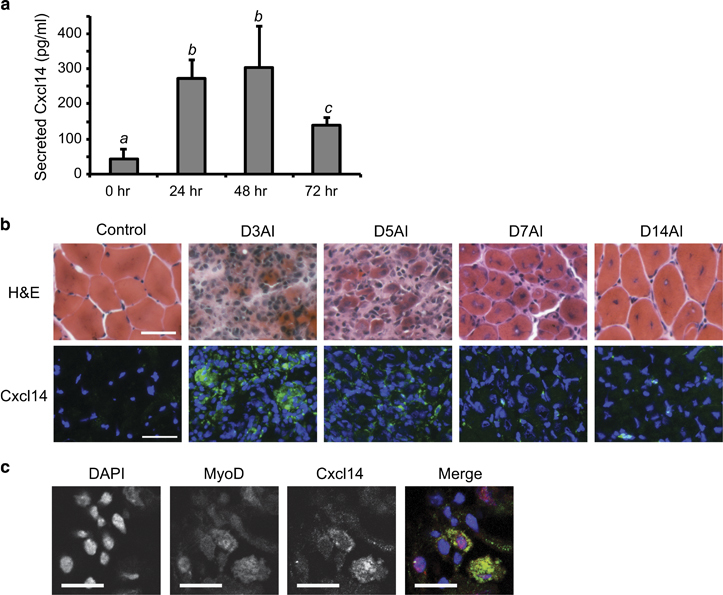
Cxcl14 is expressed in muscle cells. (**a**) C2C12 cell media over the course of differentiation (0, 24, 48, 72 h) were subjected to ELISA assay to determine secreted Cxcl14 levels (*n*=5, samples assayed in duplicates). (**b**) TA muscles were injured by BaCl_2_ injection and isolated on days 3, 5, 7 and 14 after injury (AI). Upon cryosection, H&E staining or immunofluorescence staining for Cxcl14 (green) together with DAPI (blue) was performed (*n*=3). Scale bars: 50 μm. (**c**) The procedure described in (**b**) was repeated. Muscle sections isolated on day 3 AI were immunofluorescently probed for MyoD (red), Cxcl14 (green) and DAPI (blue) (*n*=6). Scale bar: 15 μm. Paired two-tailed *t*-test was performed for the data in (**a**). The data denoted by different letters (**a**–**c**) are significantly different from each other (*P*<0.05). All error bars represent s.d. of independent replicates.

Cxcl14 emerged from our previous RNAi screen as a potential negative regulator of myogenesis.^8^ Though Cxcl14 expression has been previously noted in skeletal muscle lysates,^12,17^ no function for this cytokine had previously been suggested in muscle development or maintenance. Thus, we sought to better characterise the expression of Cxcl14 in myogenic cells. We first examined whether Cxcl14 expression changes over the course of *in vitro* differentiation using the C2C12 murine myoblast cell line. Differentiation of C2C12 cells was initiated by switching cells grown to confluence to differentiation medium containing minimum growth factors. As shown in Figure 1a, secreted Cxcl14 protein levels increased in conditioned media by ~5-fold during the first 24 h of differentiation, dropping down at 72 h.

We next evaluated the expression profile of Cxcl14 *in vivo* using a well-established model of muscle regeneration.^26,27^ Localised necrosis of the tibialis anterior (TA) muscle of the hindlimb was induced via intramuscular injection of barium chloride (BaCl_2_). Saline injection into the TA muscle of the contralateral hindlimb served as a non-injury control. We did not observe Cxcl14 signal in undamaged myofibers, though its expression was rapidly induced during the early phase of regeneration (Figure 1b). Three days after injury, Cxcl14 was observed in both damaged myofibers and mononucleated cells within the injured area, which progressively decreased over the course of regeneration. This pattern of expression was similar to that of secreted Cxcl14 protein in C2C12 cells. The presence of Cxcl14 in myofibers indicates that this cytokine is produced by muscle cells. To directly determine whether mononucleated myogenic cells expressed Cxcl14, we co-labelled injured muscles for Cxcl14 and MyoD, a marker of activated and proliferating satellite cells. As shown in Figure 1c, we observed co-localisation of Cxcl14 and MyoD in some mononucleated cells, confirming that satellite cells indeed express this cytokine during regeneration. Of note, macrophages infiltrating injured muscle tissues, marked by the pan-macrophage marker F4/80, also expressed Cxcl14 (Supplementary Figure S1).

### Cxcl14 is a negative regulator of myoblast differentiation

**Figure 2:**
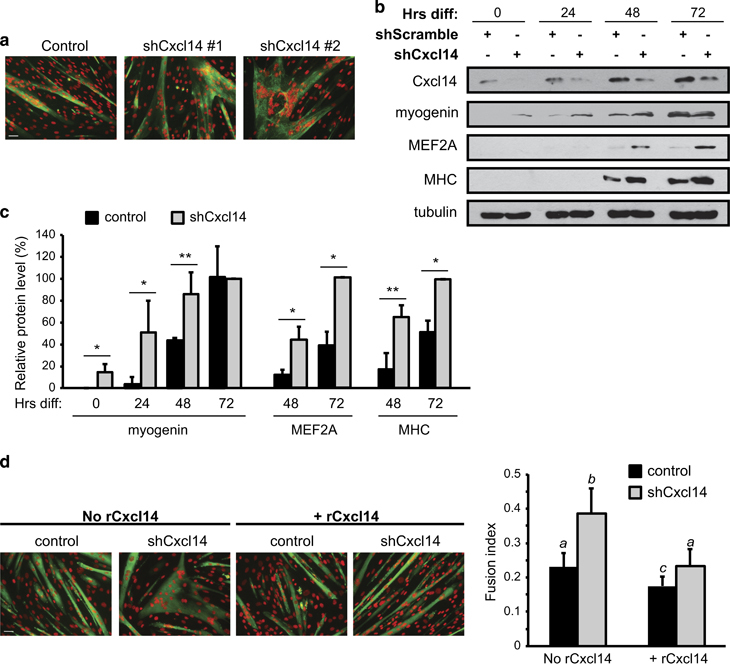
Cxcl14 knockdown enhances C2C12 differentiation. (**a**) C2C12 cells were infected with lentiviruses expressing shCxcl14 or shScramble (negative control), selected for 2 days then differentiated for 72 h, followed by staining for MHC (green) and DAPI (pseudocoloured red) (*n*=3). Scale bar: 50 μm. (**b**) Cells treated as in (**a**) were differentiated and at indicated time points of differentiation (‘Hrs diff’) were lysed and subjected to western analysis (*n*=3). (**c**) Densitometry was performed on blots in (**b**), and the relative levels of proteins using tubulin as reference were quantified. Paired *t* test was performed to compare control and shCxcl14 at each time point. *P<0.05; **P<0.01. (**d**) C2C12 cells were treated as in (**a**), then grown in the presence or absence of 25 ng/ml recombinant Cxcl14 (rCxcl14) for 24 h after selection. Cells were then differentiated for 3 days, followed by staining for MHC and DAPI and quantification of fusion index (*n*=4). Scale bar: 50 μm. Paired two-tailed *t*-test was performed for the data in (**d**). The data denoted by different letters (**a**–**c**) are significantly different from each other (*P*<0.05). All error bars represent s.d. of independent replicates.

To probe the function of Cxcl14 in myogenic differentiation, we knocked down Cxcl14 in C2C12 cells by lentivirus-delivered shRNA. We found that depletion of Cxcl14 expression by two independent shRNAs drastically increased C2C12 differentiation and fusion, leading to the formation of large, hypertrophied myotubes (Figure 2a). This increase in cell fusion was accompanied by earlier expression of muscle differentiation-specific proteins such as myogenin, MEF2A and myosin heavy chain (MHC) compared with control cells (Figure 2b and c). The expression patterns of myogenic markers during the course of C2C12 differentiation without any viral exposure were as expected (Supplementary Figure S2). The enhanced fusion index and hypertrophy phenotype observed upon Cxcl14 knockdown were reversed by adding recombinant Cxcl14 protein (rCxcl14) to the cell growth media, confirming the specificity of RNAi targeting (Figure 2d). Notably, supplementing control cells with rCxcl14 further decreased the fusion index below normal levels (Figure 2d). Taken together, these results strongly suggest that Cxcl14 functions as an inhibitor of an early step of myogenesis.

### Cxcl14 regulates myogenesis by promoting cell cycle progression

**Figure 3:**
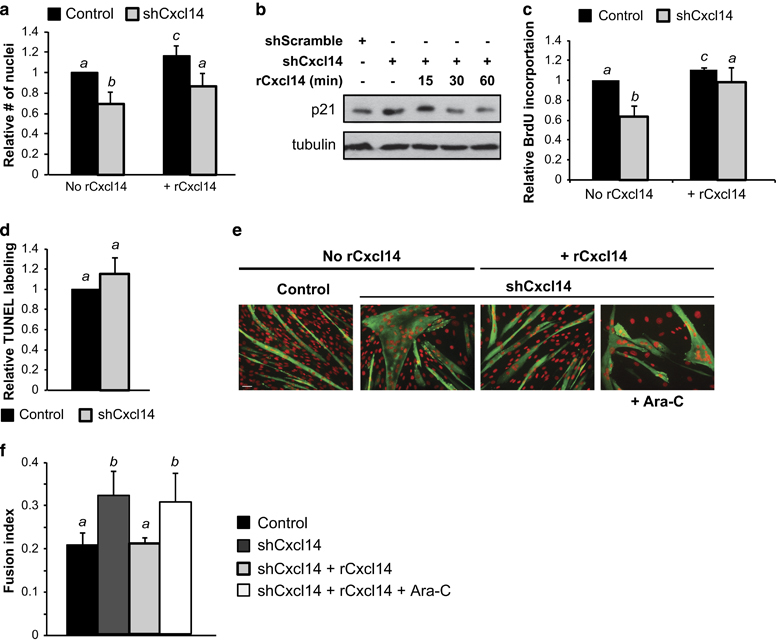
Cxcl14 promotes cell cycle progression. (**a**) C2C12 cells were infected with lentiviruses expressing shCxcl14 or shScramble, selected for 2 days, grown in the presence or absence of 25 ng/ml rCxcl14 for 24 h, followed by differentiation for 24 h and subsequent staining with DAPI. Stained nuclei were counted (*n*=4). (**b**) Cells were treated as in (**a**) but only stimulated with 10 ng/ml rCxcl14 for the indicated amount of time. Cells were then lysed and subjected to western analysis (*n*=3). (**c**) Cells were treated as in (**a**) but differentiated for 24 h, followed by BrdU incorporation for 2 h. Cells were immunofluorescently stained for BrdU and DAPI, then counted (*n*=5). (**d**) Cells were treated as in (**a**), followed by TUNEL assay to detect apoptotic cells (*n*=3). (**e** and **f**) Cells were treated as in (**a**) but grown in the presence or absence of Ara-C for 24 h, then differentiated and immunofluorescently stained for MHC (green) or DAPI (pseudocoloured red). The fusion index was calculated (*n*=3). Scale bar: 50 μm. Paired two-tailed *t*-test was performed for all the data. The data denoted by different letters (**a**–**c**) are significantly different from each other (*P*<0.05). All error bars represent s.d. of independent replicates.

In addition to enhancing expression of myogenic markers, knockdown of Cxcl14 also reduced the total number of cells present in culture (Figure 3a). Importantly, this decrease in cell number was corrected by adding rCxcl14 to the media (Figure 3a). We reasoned that the lower number of cells upon Cxcl14 knockdown could be either a result of reduced proliferation or increased cell death. Given our observation that Cxcl14 knockdown increased expression of the CDK inhibitor p21—an effect that was rapidly reversed by supplementation with rCxcl14 (Figure 3b)—we considered cell cycle regulation to be the more likely mechanism. Indeed, Cxcl14 knockdown significantly decreased proliferation in myoblasts, as indicated by a markedly lower incidence of BrdU incorporation (Figure 3c). Addition of rCxcl14 reversed the proliferation rate to normal levels in Cxcl14-depleted cells and further increased it in control cells (Figure 3c). In contrast, knockdown of Cxcl14 had no significant effect on the rate of myoblast apoptosis as measured by TUNEL labelling (Figure 3d).

We wondered whether the enhanced myogenesis phenotype upon Cxcl14 knockdown was caused simply by more rapid cell cycle withdrawal and consequently early induction of differentiation. To test this hypothesis, we repeated the cell fusion rescue experiment by knocking down Cxcl14, adding rCxcl14 to proliferating myoblasts and then differentiating the cells in the presence of the cell cycle inhibitor cytosine β-D-arabinofuranoside (Ara-C). As shown in Figure 3e and f, the introduction of Ara-C ablated the normalising effect of rCxcl14 on cell fusion. These data indicate that Cxcl14 promotes myoblast cell cycle progression, and that this function is responsible for its anti-myogenic effect.

### Erk mediates Cxcl14’s anti-myogenic effect

**Figure 4:**
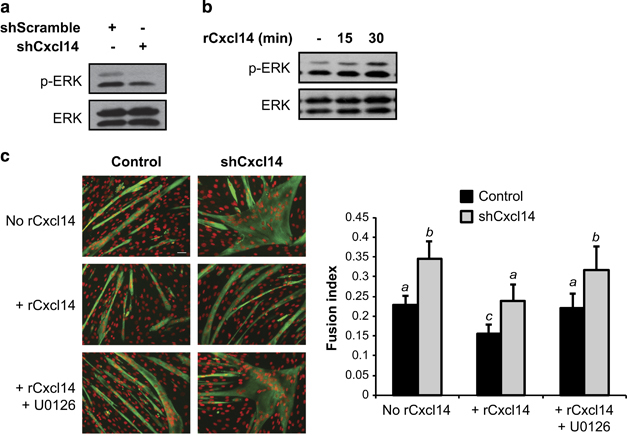
ERK1/2 mediates Cxcl14’s anti-myogenic effect. (**a**) C2C12 cells were infected with shRNA lentiviruses and selected, followed by cell lysis and western analysis (*n*=4). (**b**) Cells were treated as in (**a**), differentiated for 24 h, then stimulated with 10 ng/ml rCxcl14 for the indicated amount of time, followed by cell lysis and western analysis (*n*=3). (**c**) C2C12 cells were infected with shRNA lentiviruses as indicated, then grown in the presence or absence of 25 ng/ml rCxcl14 and U0126 for 24 h. Cells were then differentiated for 72 h and subsequently stained for MHC and with DAPI, followed by quantification of the fusion index (*n*=3). Scale bar: 50 μm. Paired two-tailed *t*-test was performed for the data in (**c**). The data denoted by different letters (**a**–**c**) are significantly different from each other (*P*<0.05). All error bars represent s.d. of independent replicates.

We next sought to understand the signalling mechanism underlying the anti-myogenic function of Cxcl14. The MAP kinase ERK1/2 has an established role in G1- to S-phase transition, thereby promoting cell proliferation,^28^ and has been reported to inhibit the initiation of myogenesis.^29^ Cxcl14 has also been linked to ERK1/2 activation in fibroblasts.^14^ Given these facts, we wondered whether Cxcl14’s pro-proliferative function was mediated through ERK1/2 signalling. Indeed, we observed reduced ERK1/2 phosphorylation in cells depleted of Cxcl14 (Figure 4a). Similarly, myoblasts exposed to rCxcl14 rapidly activated ERK1/2 (Figure 4b).

To assess the functional relevance of ERK1/2 in Cxcl14’s anti-myogenic effects, we used the pharmacological inhibitor U0126 to inhibit MEK1/2, the upstream activator of ERK1/2. As shown in Figure 4c, treatment with U0126 prior to rCxcl14 exposure prevented the normalising effect of rCxcl14 on myoblast fusion in both control and Cxcl14 knockdown cells. Taken together, these data strongly suggest that Cxcl14’s anti-myogenic function is dependent on the MEK/ERK signalling axis.

### Cxcl14 does not inhibit myogenesis via Cxcl12 antagonism

Currently, there is no established receptor for Cxcl14. One group proposed that Cxcl14 is a ligand for chemokine (C-X-C motif) receptor 4 (CXCR4) and functions as an antagonist for the cognate ligand of CXCR4, chemokine (C-X-C motif) ligand 12 (Cxcl12),^30^ although another study demonstrated that Cxcl14 does not directly modulate CXCR4 in other cell types.^31^ However, Cxcl12 is a known pro-myogenic cytokine,^4^ so Cxcl14 working as an antagonist to Cxcl12 activity seemed plausible in our model system. To test this potential relationship, we introduced recombinant Cxcl12 (rCxcl12) to proliferating cells either alone or with rCxcl14 and then induced differentiation. We observed no evidence of antagonism between the two cytokines, in either their effects on proliferation or myoblast fusion (Supplementary Figure S3). BrdU incorporation was weakly but significantly enhanced with rCxcl12 exposure, which is not surprising as this cytokine is known to activate ERK1/2 in other cell types.^32,33^ Our observation that rCxcl12 had no significant effect on cell fusion when introduced exclusively to proliferating cells is also not entirely unexpected, since Cxcl12’s established function as a regulator of second-stage fusion necessitates a later window of expression and activity.^4^ Thus, our data indicate that the anti-myogenic function of Cxcl14 is not through antagonism of Cxcl12. The identity of the Cxcl14 receptor remains elusive and warrants future research efforts.

### Cxcl14 inhibition accelerates muscle regeneration *in vivo*

**Figure 5:**
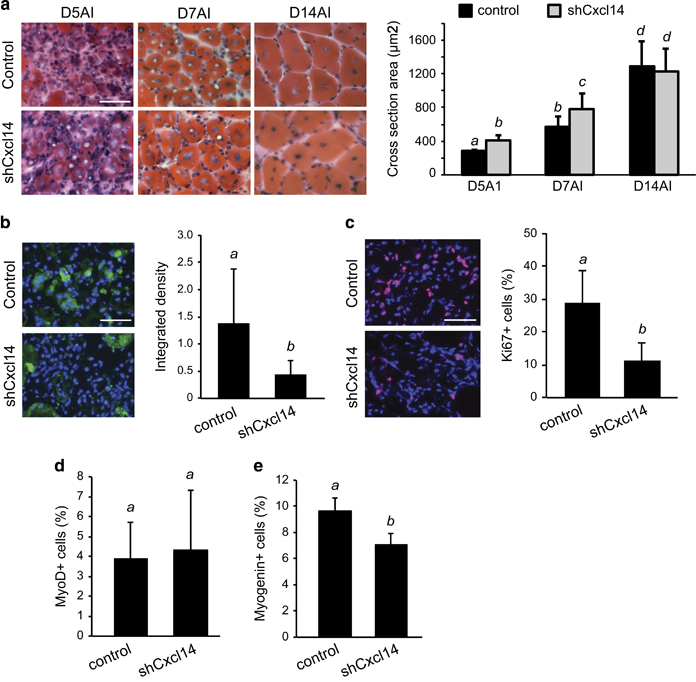
Cxcl14 knockdown accelerates muscle regeneration post-injury. (**a**) TA muscles were co-injected with BaCl_2_ and shRNA viruses, and isolated on days 5, 7 and 14 after injury (AI). Upon cryosection, H&E staining was performed and regenerating myofiber cross-sectional area (CSA) was quantified (*n*=6 for D5AI, *n*=8 for D7AI, *n*=7 for D14AI). (**b**) TA muscles injected as above were isolated on day 3 AI, cryosectioned and immunostained for Cxcl14 (green) along with DAPI (blue) (*n*=3). Fluorescence intensity was quantified using ImageJ software. (**c**–**e**) TA muscle sections as described in (**b**) were immunostained for Ki-67 (**c**), MyoD (**d**) or myogenin (**e**), and the percentage of positive cells was quantified (*n*=5). Scale bars: 50 μm. Paired two-tailed *t*-test was performed. The data denoted by different letters (**a**–**c**) are significantly different from each other (*P*<0.05). All error bars represent s.d. of independent replicates.

Given the striking enhancement of differentiation seen with Cxcl14 knockdown *in vitro* (Figure 2a and b), we wondered if manipulating the levels of Cxcl14 would have a similar effect on myogenesis *in vivo*. We utilised the same method of TA muscle injury described earlier with young adult mice, but now introduced Cxcl14 shRNA via co-injection with BaCl_2_. At days 5 and 7 after injury (AI), we observed a statistically significant increase in the size of regenerating myofibers upon Cxcl14 knockdown (Figure 5a). This difference in myofiber size disappeared a week later at day 14 AI. Effective knockdown of Cxcl14 protein by the shRNA at day 3 AI in mononucleated cells was confirmed (Figure 5b). These results indicate that suppression of Cxcl14 levels during muscle injury speeds up the normal regenerative process without changing the final myofiber size. This interpretation is in line with our *in vitro* data, which suggests that Cxcl14 inhibition simply leads to more rapid cell cycle exit, allowing for a speedier differentiation process. To further confirm this hypothesis *in vivo*, we evaluated the relative levels of dividing cells in early regeneration by probing day 3 AI muscle sections for the proliferative marker Ki-67. As expected, Cxcl14 knockdown muscles did indeed show significantly lower labelling for Ki-67, and thus lower levels of proliferation (Figure 5c). MyoD-positive cells did not decrease in number upon Cxcl14 knockdown (Figure 5d). This is not surprising because Cxcl14 regulation of cell cycle withdrawal would be expected to be downstream or independent of MyoD expression. On the other hand, the number of myogenin-expressing mononucleated cells decreased in Cxcl14 knockdown tissues (Figure 5e), which is consistent with accelerated myofiber formation and thus depletion of myogenic cells.

### Reduction of Cxcl14 expression ameliorates regenerative defect in aging muscle

Aging is accompanied by dysregulation of cytokines and increased incidence of inflammation, both at the overall organismal level and in the musculoskeletal system in particular.^34^ Similarly, it is a well-established phenomenon that muscle regenerative capacity declines with age, likely through changes in satellite cell quantity, quality and extrinsic changes to their niche.^35,36^ We wondered whether muscular Cxcl14 levels changed throughout the lifespan, and also whether this correlated to any functional defects in myogenesis. Rodent studies of aging muscle tend to focus on molecular changes and regenerative decline in very old animals (i.e., ⩾2 years old). However, we were interested in understanding when exactly muscle regenerative ability begins to decline in our mouse model.

**Figure 6:**
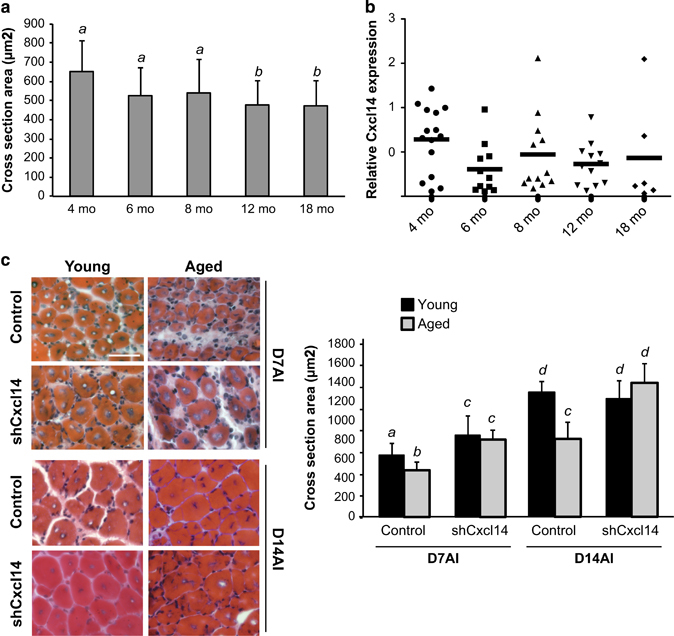
Cxcl14 enhances muscle regeneration in aging mice. (**a**) TA muscles were injured with BaCl_2_, and isolated on day 7 AI. Upon cryosection, H&E staining was performed and regenerating myofiber cross-sectional area (CSA) was quantified (*n*=12 for 4 months, *n*=9 for 6 months, *n*=11 for 8 monrhs, *n*=12 for 12 months, *n*=6 for 18 months). (**b**) TA muscle sections as described in (**a**) were isolated on day 7 AI, and the RNA was isolated and quantified by qRT-PCR. Horizontal bars represent the mean of individually plotted data points (*n*=15 for 4 months, *n*=11 for 6 months, *n*=12 for 8 months, *n*=12 for 12 months, *n*=6 for 18 months). (**c**) TA muscles from 2-month-old (young) and 12-month-old (aged) mice were co-injected with Cxcl14 or scramble shRNA viruses together with BaCl_2_, then processed as in (**a**) at day 7 and day 14 AI (*n*=6 for each). One-way ANOVA was used to analyze the data in (**b**) and paired two-tailed *t*-test was performed in (**c**). The data denoted by different letters (**a**–**c**) are significantly different from each other (*P*<0.05). All error bars represent s.d. of independent replicates. Scale bar: 50 μm.

To answer this question, we performed TA muscle injury and regeneration experiments in progressively aged mice. We used both male and female mice for these experiments as gender had no discernable effect on muscle regeneration in our experimental system (Supplementary Table S1). As shown in Figure 6a, the average size of regenerated myofibers in mice injected with BaCl_2_ began to decline at 6 months of age and the reduction of myofiber size became statistically significant by 1 year of age. We also performed qPCR to evaluate the relative mRNA levels of Cxcl14 over the aging time course. However, no significant change to Cxcl14 expression was observed (Figure 6b), indicating that normal regenerative decline in older mice is not linked to increased inhibitory signals from this particular cytokine. Nevertheless, we reasoned that knockdown of Cxcl14 in aging muscle could still recapitulate the rapid regeneration phenotype seen in young animals, or even prove therapeutic. Indeed, we observed that Cxcl14 knockdown significantly enhanced regeneration at both 7 days and 14 days AI in aged muscle, rescuing regenerating myofibers to the same size as in young mice (Figure 6c). This is in contrast to the effect of Cxcl14 knockdown in young animals, which only sped up the regeneration process and did not affect mature myofiber size at day 14 AI (Figure 5a). Taken together, these results suggest that even though Cxcl14 is not responsible for aging-induced regenerative decline, limiting its expression has the potential to therapeutically restore regeneration in aged animals.

## Discussion

We have identified Cxcl14 as a novel negative regulator of skeletal myogenesis. Our data show that Cxcl14 functions by inhibiting cell cycle exit, thereby preventing myoblast differentiation and fusion into mature myotubes. Cxcl14 has been previously implicated in promoting proliferation, particularly in cancer cells,^13,14^ though this is the first report of a role for Cxcl14 in muscle development. Interestingly, Cxcl14’s mechanism of action appears to directly counter the myogenic role of FMS-like tyrosine kinase 3 ligand (Flt3L), another cytokine that emerged from our functional RNAi screen.^8^ Further studies from our lab revealed that Flt3L promotes myogenesis by enhancing myoblast cell cycle withdrawal via suppression of ERK1/2.^9^ Considering the prolific ability of muscle cells to secrete cytokines,^5–7^ it is possible that other antagonistic pairs of cytokines exist to allow fine-tuning flexibility during muscle development. Further exploration of the network of cytokines secreted by and regulating muscle cells will likely prove very interesting for the future.

We demonstrated that inhibiting endogenous Cxcl14 has a physiologically significant effect in promoting muscle regeneration. A role for Cxcl14 in regeneration has not been previously demonstrated in any tissue. Cxcl14 expression was noted in a recent study of regenerating dental pulp,^37^ but its function was unclear. Li *et al*^38^ demonstrated that antibody-mediated neutralization of Cxcl14 protects the liver from CCl_4_-induced acute liver injury. However, they also note that Cxcl14 has an inhibitory effect on proliferation, in contrast to our results. Their findings indicate that the main effect of Cxcl14 neutralisation in the liver is prevention of necrosis and steatosis following an injury. Cxcl14’s mechanism of action in muscle regeneration appears unique and not yet found in any other tissue.

It is important to note that our regeneration studies evaluated the effect of Cxcl14 knockdown rather than complete knockout of Cxcl14 gene expression. This may be an important consideration; it stands to reason that while limiting myoblast proliferation after injury can speed up myogenesis, a severe proliferation defect in muscle progenitors may instead blunt effective muscle regeneration. It is perhaps unlikely that knockout of a single cytokine could produce such a severe proliferation defect; however, Cxcl14 knockout mice do exist and testing their muscle regenerative capacity may prove fruitful. Interestingly, Cxcl14^−/−^ mice develop normally and display no obvious phenotype, with the exception of decreased weight and feeding behaviour, though a disturbed Mendelian breeding pattern and occasional postnatal death of newborns at day 2–3 was noted.^25,39^ The authors make no mention of perturbations to the development or regeneration of skeletal muscle; since Cxcl14 had no previous functional link to myogenesis, it is possible that these processes were not evaluated. Nonetheless, transient delivery of shRNA may offer an advantage over systemic Cxcl14 knockout as it avoids compensatory effects that are likely to occur when a gene is knocked out during development.

It is possible that the enhanced cell cycle withdrawal observed with Cxcl14 inhibition could prove therapeutic for muscle diseases. For example, recent studies of Duchenne muscular dystrophy have revealed that activated dystrophin-deficient satellite cells have a prolonged cell cycle and defective asymmetrical division, leading to abnormally high numbers of quiescent satellite cells and lower numbers of myoblasts capable of differentiation.^40,41^ Intriguingly, one microarray study noted higher levels of Cxcl14 mRNA in the muscles of DMD model mdx mice compared with control mice.^42^ While the pathogenesis of muscular dystrophy is multifaceted, it would be interesting in future studies to re-evaluate the functional significance of Cxcl14 depletion in the context of this disease.

Another disease linked to cytokine dysregulation is sarcopenia, or the progressive loss of muscle mass that comes with advancing age. Sarcopenia, which affects ~50% of people over the age of 80, occurs in the absence of underlying illness and is correlated with physical frailty.^34,43–45^ Our current understanding of sarcopenia places chronic inflammation as a key player in the progression of this disease.^34,45^ And yet, still very little is known about the effects of cytokines on skeletal muscle tissue under normal physiological conditions or pathological states. Strikingly, we have observed that the impaired regenerative capacity in aging muscle is fully restored by Cxcl14 depletion, suggesting a potential therapeutic strategy against sarcopenia. Although Cxcl14 expression did not significantly change over the course of our aging study, it is possible that other muscle-derived cytokines do become dysregulated over time. A deeper understanding of the network of cytokines involved in the maintenance of muscle mass—as well as how those cytokines are regulated—could shed some light on why muscle catabolism increases with age and how to prevent it.

## Materials and methods

### Antibodies and other reagents

Anti-MHC (MF20) and anti-myogenin (F5D) were obtained from the Developmental Studies Hybridoma Bank developed under the auspices of the NICHD, National Institutes of Health and maintained by The University of Iowa, Department of Biological Sciences. Anti-Cxcl14 (NBP1–31398) and anti-MyoD (NB100–56511) were from Novus Biologicals (Littleton, CO, USA). Anti-F4/80 (MCA497RT) was from Bio-Rad (Hercules, CA, USA). Anti-tubulin (ab11304) was from Abcam (Cambridge, MA, USA). Anti-p21 (sc-471:M-19) was from Santa Cruz Biotechnology (Dallas, TX, USA). All other primary antibodies were from Cell Signaling Technology (Danvers, MA, USA): anti-ERK: no. 9102; anti-pERK: No. 9106; anti-MEF2a: No. 9736. Alexa Fluor fluorescent secondary antibodies were from Thermo Fisher Scientific (Waltham, MA. USA). All other secondary antibodies were from Jackson ImmunoResearch Laboratories, (West Grove, PA, USA). Gelatin, BrdU and AraC were from Sigma-Aldrich (St. Louis, MO, USA). Recombinant Cxcl14 protein was from Novus Biologicals. U0126 and ELISA kit for detection of mouse Cxcl14 was from Thermo Scientific.

### Cell culture

C2C12 cells were a gift from the SJ Kaufman lab at the University of Illinois and originally obtained from ATCC. They are routinely tested negative for mycoplasma contamination in our laboratory (most recent testing in August 2016). Cells were maintained in DMEM (4.5 g/l glucose) supplemented with 10% fetal bovine serum and 1% penicillin-–treptomycin at 37 °C with 7.5% CO_2_. To induce differentiation, cells were plated on tissue culture plates coated with 0.2% gelatin and grown to 100% confluence before switching to differentiation medium (DMEM containing 2% horse serum). The cells were replenished with fresh differentiation medium daily for 3 days.

### Immunofluorescence microscopy and quantitative analysis of myocytes

C2C12 cells differentiated in 12-well plates were fixed and stained for MHC, and with DAPI as previously described.^46^ Cells were examined with a Leica DMI 4000B fluorescence microscope (Leica, Wetzlar, Germany). The fluorescent images were captured using a RETIGA EXi camera (QImaging, Surry, BC, Canada) and Image Pro Express software (Media Cybernetics, Rockville, MD, USA). Images were analyzed using ImageJ software (NIH, Bethesda, MD, USA). The fusion index was calculated as the percentage of total nuclei in myotubes (cells with 2 or more nuclei). Each data point was generated from quantifying all cells in five randomly chosen microscopic fields, totaling 2000–3500 nuclei.

### Lentivirus-mediated RNAi

shRNAs in the pLKO.1-puro vector were purchased from Sigma-Aldrich (MISSION TRC). Clone IDs are: shCxcl14 No. 1, TRCN0000065369; shCxcl14 No. 2, TRCN0000065370. A hairpin of scrambled sequence (shScramble) as a negative control and lentivirus packaging were previously described.^9^ Virally transduced C2C12 cells were selected in 3 μg/ml puromycin for 2 days, followed by differentiation in media containing puromycin for 3 days.

### Quantitative RT-PCR

C2C12 cells or regenerating muscles were lysed in Trizol (Invitrogen, Carlsbad, CA, USA), and RNA was isolated following the manufacturer's protocol. RNA was isolated from frozen regenerating muscles using the mirVana miRNA isolation kit from Thermo Fisher Scientific. cDNA was synthesized from 1 μg RNA using the RealMasterScript SuperMix cDNA synthesis kit (5Prime, Hilden, Germany) following the manufacturer's protocol, followed by quantitative PCR on a StepOne Plus (Applied Biosystems, Foster City, CA, USA) using gene-specific primers. β-actin was used as a reference to obtain the relative fold change for target samples using the comparative C_T_ method. Mouse β-actin primers: forward 5′- ttgctgacaggatgcagaag-3′; reverse 5′- atccacatctgctggaaggt-3′. Mouse Cxcl14 primers: forward 5′- ggtccaagtgtaagtgttcc-3′; reverse 5′- cctggacatgctcttggtg-3′.

### Western blotting

Cells were lysed in SDS sample buffer with 10% β-mercaptoethanol. Proteins were resolved by SDS-PAGE and transferred onto polyvinylidene fluoride (PVDF) membrane (EMD Millipore, Darmstadt, Germany) and incubated with various antibodies following the manufacturers’ recommendations. Detection of horseradish peroxidase-conjugated secondary antibodies was performed with chemiluminescence solution (100 mmol/l Tris-HCl, 0.009% H_2_O_2_, 225 μmol/l coumaric acid, 1.25 mmol/l luminol) and developed on X-ray films. Quantification of western blot band intensities was performed by densitometry of X-ray images using ImageJ software (NIH).

### Cell proliferation and apoptosis assays

To measure proliferation of C2C12 cells, BrdU labelling was performed as previously described.^9^ To assess apoptosis, TUNEL assays were performed following manufacturer’s manual (Promega, Madison, WI, USA).

### Injury-induced muscle regeneration and manipulation of Cxcl14 expression in mice

Male FVB mice aged 8–10 weeks were used in all the regeneration experiments, unless otherwise indicated. Male and female FVB mice of various ages (4, 6, 8, 12 or 18 months old) were used in the aging muscle experiments. Animals were randomly selected for experiments at the appropriate ages, and all experiments were performed identically. Muscle injury was induced by injection of BaCl_2_ (50 μl of 1.2% w/v in saline) into TA muscles as previously described.^27^ On various days after injury, the mice were killed and the TA muscles were collected, followed by RNA extraction or cryosection and staining. To knockdown Cxcl14, shCxcl14 viruses (and shScramble as control) as described above, but concentrated to 100× via ultracentifugation, were co-injected with BaCl_2_ into mouse hind limb TA muscles. The injected muscles were collected 5, 7 or 14 days after injury and subjected to RNA isolation or cryosection. Samples were excluded from analysis, if the extent of muscle injury was drastically lower than that typically observed.

### Muscle tissue cryosection, hematoxylin and eosin staining, and immunohistochemistry

TA muscles were isolated, frozen in liquid-nitrogen-cooled 2-methylbutane and embedded in TBS tissue freezing medium (Thermo Fisher Scientific). Sections of 10 μm thickness were obtained with a cryostat (Microm HM550; Thermo Fisher Scientific) at −20 °C, placed on uncoated slides, and stained with hematoxylin and eosin (H&E). Separately, the sections were fixed by 1.5% paraformaldehyde, incubated with anti-Cxcl14, Ki-67, MyoD, myogenin or F4/80 antibody, followed by incubation with Alexa-conjugated secondary antibodies and DAPI. Imaging was performed with a fluorescence microscope (DMI 4000B; Leica) with a 20× dry objective (Fluotar, numerical aperture 0.4; Leica). Quantification of fluorescence signals, when applicable, was performed using ImageJ software. The bright field and fluorescence images were captured at 24 bit and 8 bit, respectively, at room temperature using a camera (RETIGA EXi; QImaging,) equipped with Image Pro Express software (Media Cybernetics). Visualisation of MyoD and Cxcl14 co-localisation in regenerating muscle was achieved using a confocal microscope (Carl Zeiss LSM700, Oberkochen, Germany) with a 40× oil-immersion objective and Zen software (Zeiss). The images were then processed in Photoshop CS5 (Adobe, San Jose, CA, USA) where brightness and contrast were adjusted. Fluorescence images were pseudocoloured and adjusted, when necessary, by identical parameters for all samples in the same experiment. A total area of 464 000 μm^2^ from the degenerated regions of each TA muscle was scored for centrally nucleated regenerating myofiber numbers and their cross-sectional area (CSA) using ImageJ software. Each data point was generated from quantifying all regenerating fibers in three randomly chosen microscopic fields within the injured area. Intramuscular injection, tissue processing and the data analysis were performed by multiple researchers in order to minimize subjective bias. No blinding was performed.

### Statistics

All the data shown are representative results of at least three independent experiments in cells, or 3–15 mice for each data point in animal experiments. The exact sample size for each experiment is described in Figure legends. All the quantitative data are presented as mean±s.d from independent experiments. Whenever necessary, statistical significance of the data comparison was analyzed by performing Student’s *t*-tests or ANOVA as indicated in the Figure legends. The data were tested graphically for normality. Variation within each group was assessed to ensure variance was comparable between the groups of statistical comparison.

### Study approval

All animal experiments in this study followed protocols approved by the Animal Care and Use Committee at the University of Illinois at Urbana-Champaign.

## The paper explained

### Problem

The complex signalling mechanisms underlying skeletal muscle maintenance and regeneration are still not fully understood, although dysregulation of these processes contributes to the progression of muscle diseases such as cachexia, sarcopenia and the suite of muscular dystrophies.

### Results

The cytokine Cxcl14 is an endogenous inhibitor of myoblast differentiation and skeletal muscle regeneration. Reducing Cxcl14 expression in muscle tissue after injury accelerates the regeneration process in young adult mice, and more importantly, fully rescues regeneration capacity in aging mice with impaired regenerative response. Cxcl14 functions in myoblasts to prevent cell cycle withdrawal and subsequently myogenic differentiation. This effect occurs through activation of the pro-proliferative MAP kinase ERK1/2.

### Impact

Inhibition of Cxcl14 expression may represent a new approach to prevent muscle wasting after injury or in the aging population. Moreover, these results add Cxcl14 to the small family of muscle-derived cytokines with a known function in myogenesis. It is possible that a network of secreted factors governs many of the steps of myoblast differentiation, and elucidating the roles of these factors can deepen our understanding of muscle disease and reveal novel therapeutic targets.
