# Supplementary material for: Cxcl14 depletion accelerates skeletal myogenesis by promoting cell cycle withdrawal
Source: NPJ Regen Med. 2017 Jan 5;2:16017–. doi: 10.1038/npjregenmed.2016.17 (PMC5537738; doi:10.1038/npjregenmed.2016.17)
Supplement: Supplementary Information [file npjregenmed201617-s1.pdf]

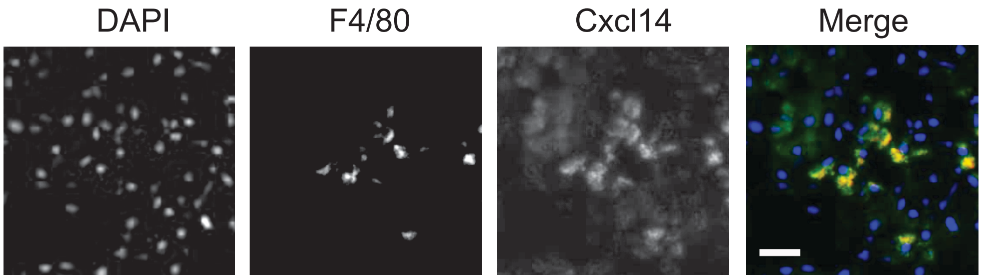

Fig. S1. Macrophages express Cxcl14

TA muscles were injured by $BaCl_2$ injection, isolated on days 3 AI, cryosectioned, and immunostained for Cxcl14 together with F4/80 and DAPI. The merged image was pseudo-colored as follows: DAPI, blue; F4/80, red; Cxcl14, green. n=3. Scale bar: 15 μm.

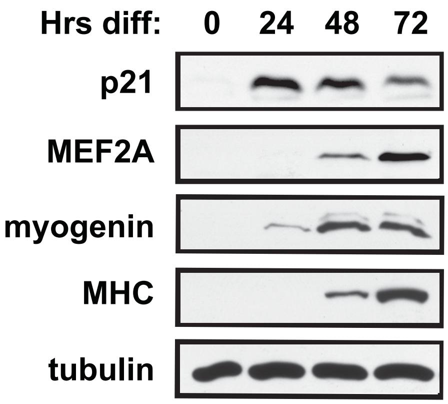

Fig. S2. Expression of myogenic markers in C2C12 differentiation

C2C12 myoblasts were induced to differentiate by switching to low-serum medium. At the indicated time points cells were lysed and subjected to western analysis.

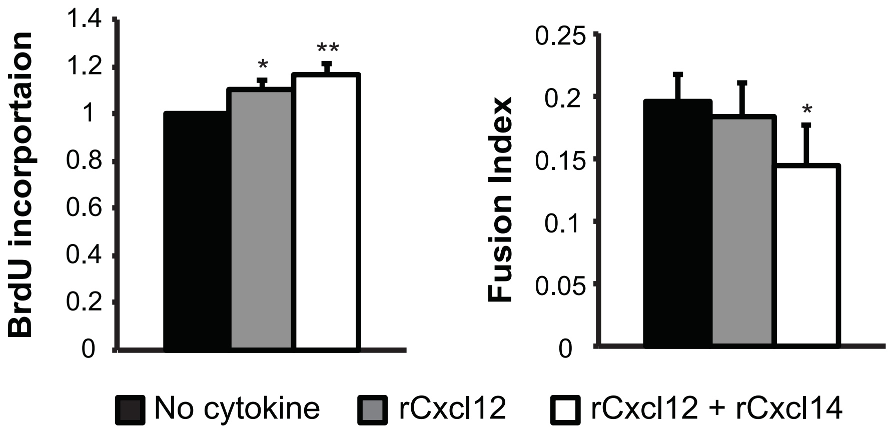

Fig. S3. Cxcl14 does not antagonize Cxcl12 in the early stage of myogenesis

C2C12 cells were grown in the presence or absence of rCxcl12 and rCxcl14 for 24 hrs, then differentiated 24 hrs followed by BrdU incorporation (n=3). Alternatively, cells were differentiated for 72 hrs after cytokine exposure and subsequently stained for MHC and with DAPI (n=4). The number of BrdU+ cells and the fusion index were quantified.

Paired 2-tailed t test was performed. Different letters indicate statistically significant difference (*P*<0.05). All error bars represent SD of independent replicates.

Table S1. Gender of mice does not affect muscle regeneration

TA muscles of 8-10 week-old female FVB mice were injured with $BaCl_2$, and isolated on day 7 AI or day 14 AI (n=5 for each time point). Upon cryosection, H&E staining was performed and regenerating myofiber cross-sectional area (CSA) was quantified. When compared to age-matched male FVB mice (Ge et al., 2009; n=7 for each time point), no significant difference was observed in the regenerating myofiber CSA in females. Paired 2-tailed t test was performed to evaluate statistical significance.

	**D7AI (CSA)**		**D14AI (CSA)**	
	**Males**	**Females**	**Males**	**Females**
**Mean**	804 μm^2	787 μm^2	1425 μm^2	1568 μm^2
**Standard Deviation**	+/- 96 μm^2	+/- 165 μm^2	+/- 176 μm^2	+/- 24 μm^2
***P*-value**	0.92		0.14	
